# Driving fatigue increases after the Spring transition to Daylight Saving Time in young male drivers: A pilot study

**DOI:** 10.1016/j.trf.2023.10.014

**Published:** 2023-11

**Authors:** Federico Orsini, Gianluca Giusti, Lisa Zarantonello, Rodolfo Costa, Sara Montagnese, Riccardo Rossi

**Affiliations:** a Department of Civil, Environmental and Architectural Engineering, University of Padua, Padua, Italy; b Mobility and Behavior Research Center – MoBe, University of Padua, Padua, Italy; c Department of General Psychology, University of Padua, Padua, Italy; d Department of Medicine, University of Padua, Padua, Italy; e Chronobiology Section, Faculty of Health and Medical Sciences, University of Surrey, Guildford, United Kingdom; f Institute of Neuroscience, National Research Council (CNR), Padua, Italy; g Department of Biology, University of Padua, Padua, Italy

**Keywords:** Daylight saving time, Driving fatigue, Driving simulator, Circadian desynchrony, Sleep deprivation

## Abstract

The Spring transition to Daylight Saving Time (DST) has been associated with several health and road safety issues. Previous literature has focused primarily on the analysis of historical crash and hospitalization data, without investigating specific crash contributing factors, such as driving fatigue. The present study aims to uncover the effects of DST-related circadian desynchrony and sleep deprivation on driving fatigue, by means of a driving simulator experiment.

Eighteen participants (all males, age range 21–30 years, mean = 24.2, SD = 2.9) completed two 50-minute trials (at one week distance, same time and same day of the week) on a monotonous highway environment, the second one taking place in the week after the Spring transition to DST. Driving fatigue was evaluated by analysing several different variables (including driving-based, physiological and subjective indices) and by comparison with a historical cohort of pertinent, matched controls who had also undergone two trials, but in the absence of any time change in between.

Results showed a considerable rise in fatigue levels throughout the driving task in both trials, but with significantly poorer performance in the post-DST trial, documented by a worsening in vehicle lateral control and an increase in eyelid closure. However, participants seemed unable to perceive this decrease in their alertness, which most likely prevented them from implementing fatigue-coping strategies. These findings indicate that DST has a detrimental effect on driving fatigue in young male drivers in the week after the Spring transition, and provide valuable insights into the complex relationship between DST and road safety.

## Highlights

• Vehicle lateral control and eyelid closure were negatively affected by the DST transition.
• Drivers less accustomed to managing sleepiness were impacted the most.
• Participants were substantially unaware of their decrease in alertness.
• Their consequent inability to implement fatigue-coping strategies may lead to road safety risks.

## 1 Introduction

In terms of human physiology, Daylight Saving Time (DST) represents a situation of conflict between the temporal information that our cerebral master circadian clock acquires and interprets from the environment (i.e. light-dark cues, or the solar time of the place we live in) and the time we arbitrarily decide to set on our clocks (which encompasses both times zones and DST) (Roenneberg, Winnebeck, & Klerman, 2019). Such conflict (or desynchrony) is not limited, as it has been often suggested, to the few days around the Spring transition to DST, but it is maintained all through the DST months (Ferrazzi et al., 2018), with negative consequences on sleep quality and sleep duration, and thus on daytime performance.

The available published literature on road safety in relation to the transitions to and from DST has been mostly based on retrospective analyses of historical crash data and has been generally focused on the days/weeks around the transitions. Within this frame, it has been difficult to isolate any specific crash contributors such as drivers’ vigilance, time of day, lighting versus traffic conditions etc., which has resulted in somewhat heterogeneous and conflicting results (Coate and Markowitz, 2004, Ellis et al., 2016, Lahti et al., 2010, Robb and Barnes, 2018, Singh et al., 2022, Sullivan and Flannagan, 2002).

These contradicting results are likely to depend on the competing effects of different factors. On the one hand, increases in the number of road crashes after the transition to DST have been attributed to sleep deprivation and circadian misalignment (Fritz, VoPham, Wright, & Vetter, 2020); on the other hand, the daytime-dependent illumination change which improves visibility during afternoon traffic peak-hours has been associated with a reduction in road crashes after DST (Huang & Levinson, 2010). According to a review by Carey and Sarma (2017), sixteen studies had investigated the short-term effect of DST on road safety, with inconsistent results: 19% reported a crash reduction, 38% a crash increase, 44% no significant changes. More recently Zhou and Li (2022) have also reported contrasting results in their review of the literature. Therefore, several fundamental questions regarding the effects of the Spring transition to DST on road safety remain open.

Orsini, Zarantonello, Costa, Rossi, and Montagnese (2022) have proposed an alternative approach, which, instead of analysing aggregated crash counts, investigated changes in specific driving behaviours, such as the propensity towards overtaking violations, reaction times at signalized intersections, safety distance on overtaking cyclists and exiting manoeuvres from freeways. This was achieved by means of a driving simulator experiment in which participants underwent two trials, before and after the Spring transition to DST, and uncovered significant DST-related driving performance impairment, resulting in riskier behaviour, impinging on reaction times and situation awareness, and on the ability to modify behaviour based on recently acquired information. Due to the relatively short duration of the driving tasks in the experimental design of Orsini et al. (2022), it was not possible to study the effects of the DST transition on driving fatigue, which is particularly relevant to road safety and worthy of further investigation.

Indeed, driving fatigue and sleepiness are among the most important contributing factors to road crashes (Bioulac et al., 2017, Gonçalves et al., 2015), accounting for up to 21% of total fatal crashes (Bayne et al., 2022). The two terms, which are often used interchangeably in the literature, actually have different definitions and causes, but their effects are similar in terms of decrease in physical and mental performance (Observatory, 2021, Phillips, 2014).

According to May and Baldwin (2009), driving fatigue can be divided into sleep-related (SR) and task-related (TR) fatigue. The former can be caused by sleep deprivation and circadian desynchrony, and the latter by the driving task and the environment. TR fatigue can be further subdivided into active and passive fatigue, which relate to conditions of mental over- and under-load, respectively (Gimeno, Cerezuela, & Montañés, 2006). The investigation of driving fatigue is carried out by driving simulator experiments (Åkerstedt et al., 2005, Boyle et al., 2008, Wörle et al., 2021) or naturalistic studies (Philip et al., 2005), i.e. by inducing sleepiness by varying degrees of sleep deprivation (Jongen, Perrier, Vuurman, Ramaekers, & Vermeeren, 2015), by comparing performance at different circadian times (Sandberg et al., 2011), or both (Du, Zhao, Zhang, Zhang, & Rong, 2015). Most of these experiments require participants to perform a driving task in a monotonous environment, therefore combining SR and passive TR fatigue in an effort to enhance the resulting effects (May & Baldwin, 2009). Such combination is more common and thus more relevant to the highway scenario (Lyznicki, Doege, Davis, Williams, & Bresolin, 1998).

The focus of the present study was to investigate the effect of the Spring transition to DST on driving fatigue in young male drivers in a monotonous highway environment. Here, by driving fatigue, we refer to a combination of passive TR fatigue (which relates to the monotonous driving task) and SR fatigue (which relates to DST); the experimental design chosen allowed us to isolate these two components.

As evidenced by the contrasting results reported in the literature, it is not possible to establish the relative role of different DST-related factors (e.g., sleep deprivation, changes in ambient lighting etc.) with an analysis relying solely on crash data. Therefore, as already suggested by our group (Orsini et al., 2022), alternative approaches are necessary. The driving simulator experiment designed in this study allowed us to focus on one relevant factor, which is the sleep-related effect of DST on driving fatigue. Since driving fatigue is one of the most serious contributors to road crashes, in particular for young male drivers (Li et al., 2017, Martiniuk et al., 2013, Zhang et al., 2016), and since it has been shown that young drivers are more prone to driving impairment as a consequence of sleep deprivation (Cai et al., 2021), investigating this specific issue on a group of young males seems relevant. To the best of our knowledge, this represents the first study focusing on DST-related effects on driving fatigue.

## 2 Methodology

### 2.1 Driving simulator experiment

#### 2.1.1 Participants

Twenty participants, all males were recruited. Two of them were unable to return for the second experimental session and were excluded from the analysis. The resulting sample had an age range of 21–30 years (mean = 24.2, SD = 2.9). All participants had normal or corrected-to-normal vision, but not with glasses, due to incompatibility with the eye tracker used (*vide infra*). They had at least one year of driving experience with a full license and a minimum 1,000 km of average annual mileage. None had previous experience with driving simulators, significant diseases, or sleep disorders; they were not on psychoactive medication or other medication known to affect sleep.

The choice of exclusively investigating male subjects was motivated by the fact that previous studies have shown different effects of sleep deprivation on cognitive performance in males compared to females (Alhola and Polo-Kantola, 2007, Alvaro et al., 2018), and the strict time constraints of the experimental procedure (please refer to Section 2.1.3 here below) were not compatible with a large enough sample size to include an adequate number of both males and females.

Participants received monetary compensation for completing the experiment and were naïve to its aim. The experimental protocol was approved by the Ethics Committee for Psychological Research of the University of Padua (ID C7ACFE5F4436C27AC5CA25ABD8F68129), in compliance with the Code of Ethics of the World Medical Association (Declaration of Helsinki, World Medical Association, 2013). Written informed consent was obtained from all participants.

Part of a historical cohort (*vide infra*) from a previous driving simulator experiment (Gastaldi, Rossi, Hadas, Fasan, Keren, & Mulatti, 2016) was utilized as a control group to account/correct for any learning effects (i.e. differences in the observed indices of driving fatigue between the two trials being related to familiarization with the test rather than to DST). The historical cohort participants had also undergone two driving trials (with one-, two-, or three-week distance from the first trial), in exactly the same monotonous highway scenario, for 50 min (Gastaldi et al., 2016). Of these 72 total participants, 15 were males (age range 21–34 years, mean = 24.2, SD = 3.4) returning for the second trial exactly one week after the first, and without any DST transition in between, and were therefore chosen as a final reference group. This group was similar to the experimental one in terms of demographics, driving experience/frequency and sleep-wake habits (Table S1 of the Supplementary materials). No eye tracker data were available for the control group.

#### 2.1.2 Apparatuss

**Fig. 1:**
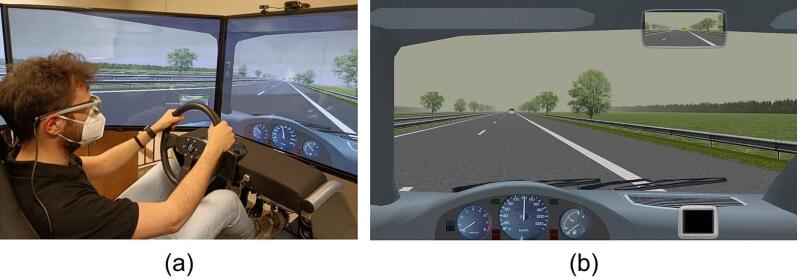
Driving scenario: (a) driving simulator setup; (b) virtual environment viewed by the participant.

The Mobility & Behaviour Research Center (MoBe) dynamic driving simulator with 2 degrees of freedom was used for the experiment (Fig. 1a). The simulator has been previously validated (Rossi et al., 2020, Rossi et al., 2014) and used in several road safety studies (Orsini et al., 2022, Orsini et al., 2019, Rossi et al., 2021, Rossi et al., 2012, Rubaltelli et al., 2021). The software was produced by StSoftware (the Netherlands); the hardware consisted of a cockpit with an adjustable seat, a force-feedback steering wheel, three pedals, and manual gearbox; the cockpit was surrounded by five 60-inch full-HD screens (creating a 330° by 45° field of view) and six speakers (three in the front, two in the rear, plus a subwoofer). The simulation system collected thirty-one vehicle kinematic variables with a sampling rate of 50 Hz.

Eye gaze was recorded throughout the driving task using the SMI eye-tracking glasses 2 Wireless (SensoMotoric Instruments, Germany), which is a non-invasive system designed similarly to a common pair of glasses and equipped with HD cameras (resolution 1280 × 960p), collecting binocular information regarding gaze position and eye activity. Data were sampled at 60 Hz and extracted using BeGaze software (SensoMotoric Instruments, Germany).

#### 2.1.3 Experimental design and procedure

The experiment consisted of two 50-minute driving simulator trials on the same virtual environment (Section 2.1.5) performed in two different days, separated by one week exactly. The first trial took place in the week preceding the switch to DST (March 21st – 25th, 2022), the second one in the week after (March 28th – April 1st, 2022). Experiments were carried out in the afternoon, from Monday to Friday; four trials were carried out each day, the first starting at 15:00 and the last at 18:00. This time window was chosen to mitigate the potential effects of the “post-lunch dip” on driving fatigue (Zhang, Yan, Wu, & Qiu, 2014), and consequent possible SR confounding effects, and to align the present experiment to that of the historical cohort (Gastaldi et al., 2016). For each participant, the two trials took place on the same weekday, at the same time. Due to these strict constraints, the experimental group could encompass a maximum of 20 participants.

In the days preceding the first trial, participants were asked to complete several online questionnaires regarding their sociodemographic, sleep quality, sleep timing, and chronotype features. On trial days, perceived sleepiness and fatigue were recorded before and after the driving simulator test, using the Stanford Sleepiness Scale (SSS, Hoddes, Zarcone, Smythe, Phillips, & Dement, 1973) and the Samn-Perelli Fatigue Scale (SPF, Samn & Perelli, 1982), respectively. Participants were also asked information on sleep onset/wake-up time on the night immediately before the test. Finally, during the trial, participants were asked to self-assess their sleepiness by verbally reporting it every 9 min, using a modified version of the Karolinska Sleepiness Scale (KSS, Åkerstedt et al., 2010), which ranges between from 1 (“not at all”) to 10 (“extremely sleepy”). Please refer to Section 2.1.4 here below for further details. The 9-minute time interval was chosen as a compromise between the need to have enough data points to track the evolution of KSS scores throughout the driving task and the need to limit as much as possible the presence of stimuli which could arouse the participants.

The driving task consisted of driving for 50 min on a monotonous highway environment, the features of which are described in Section 2.1.5. Participants were instructed to drive as they normally would in a natural setting, and to verbally report their KSS score when a question mark appeared on a virtual dashboard monitor placed on the lower right corner of the screen (Fig. 1b). The score was manually recorded by the operator. During the driving task, the question mark was shown every 9 min, i.e., five times in total, and remained on the screen for 5 s; in the few cases in which the participants did not respond, the response was qualified as missing. The question mark was positioned in a peripheric area of the field of view to minimize participant's activation. The potential distracting effect of these 5 brief periods of time during which the participants were effectively performing a divided attention task is likely to have been negligible when analysing data aggregated in 5-minute epochs (please refer to Section 2.3).

No information on the duration of the trial or the frequency of the sleepiness evaluation was provided beforehand; participants were asked to turn off their phones and remove any wristwatches before starting.

An average temperature between 20 °C and 22 °C, and a 4-lx illuminance were maintained in the driving simulator room.

#### 2.1.4 Sleep-wake assessment

The following questionnaires were administered prior to the first trial:

*The Sleep Timing Sleep Quality Screening (STSQS) questionnaire* (Montagnese, Middleton, Skene, & Morgan, 2009), which provides an assessment of sleep quality rated on a 0–10 visual-analogue scale (0 = worst, 10 = best sleep ever) plus collection of information on habitual sleep timing, including bedtime, try to sleep time, sleep latency, wake up and get up time.

*The Pittsburgh Sleep Quality Index (PSQI)* (Buysse et al., 1989, Curcio et al., 2013). Responses to 19 questions are used to generate seven components (covering subjective sleep quality, sleep latency, sleep duration, sleep efficiency, sleep disturbance, use of sleep medication, daytime dysfunction), each of which is scored from zero (best) to three (worst). These scores are then summated to provide the total PSQI score (range: 0–21); scores of >5 identify ‘poor sleepers’ (Buysse et al., 1989).

*The Epworth Sleepiness Scale (ESS)* (Johns, 1991, Vignatelli et al., 2003). Subjects rate their likelihood of ‘dozing off’ in eight different day-time situations, on a scale of zero (unlikely) to three (very likely). The total score ranges from 0 to 24 and scores of ≥11 indicate excessive daytime sleepiness (Johns, 1991).

*The self-morningness/eveningness (Self-ME) questionnaire* (Turco et al., 2015). This is a single-question assessment of chronotype through which participants qualify themselves as definitely morning, morning, evening or definitely evening types. In this specific instance, participants were then qualified as morning (grouping definitely morning and morning) or evening (grouping definitely evening and evening) for power reasons.

*The ultra-Short version of the Munich ChronoType Questionnaire (µMCTQ)* (Ghotbi et al., 2020). This is a 6-question adaptation of the MCTQ (Roenneberg, Wirz-Justice, & Merrow, 2003), which allows for quick assessment of sleep duration, midsleep (i.e. the midpoint, expressed as clock time, between sleep onset and sleep offset on free and work/study days) and social jetlag (in this instance, the uncorrected difference between midsleep on free and work/study days).

The following questionnaires were administered on trial days, before and after the driving task:

*The Stanford Sleepiness Scale (SSS)* (Hoddes et al., 1973)*.* This is a one-item self-reported questionnaire measuring subjective sleepiness on a 7-point scale, with scores >3 qualifying the participant as “sleepy” (Berry & Wagner, 2014).

*The Samn-Perelli Fatigue Scale (SPF)* (Samn & Perelli, 1982). This is a one-item self-reported questionnaire measuring subjective fatigue, originally developed to assess the fatigue of aircraft pilots, but commonly applied to driving (Hamid et al., 2016, Hilditch et al., 2017). It ranges from 1 (“fully alert, wide awake”) to 7 (“completely exhausted, unable to function effectively”). An SPF score of 5 is considered a critical threshold in civil aviation (CAA SRG, 2005).

#### 2.1.5 Driving scenario

Participants were asked to drive on a monotonous highway scenario, which consisted of a dual carriageway 164 km long road segment. Each carriageway had two driving lanes (width 3.75 m) and a hard shoulder (width 2.50 m) with a virtual bank that created a slight tendency to deviate to the right, requiring drivers to be alert enough to make compensatory steering corrections to keep the vehicle within the carriageway. The posted speed limit was 130 km/h. Trees were placed on both sides of the road and to close the line of vision on the horizon (Fig. 1b). Daytime and cloudy weather conditions, which allowed good visibility (up to 500 m) were adopted. There were no wind effects on the lane.

In both directions, very light traffic conditions were simulated (about 300 vehicles per hour per lane in the main direction, 350 in the opposite direction). In the main direction, traffic was composed of vehicles moving at equal or higher speed than that of the participant, so that participants could not perform active overtaking manoeuvres, thus further contributing to mental underload.

In order not to interrupt the driving task, even in the case of a crash, the participant’s vehicle could compenetrate both other simulated vehicles and safety barriers, while the simulation system recorded the occurrence of all events.

### 2.2 Variables analysed

Driving fatigue was investigated considering different types of metrics/indices: an objective driving-based measure (SDLP), an objective physiological measure (PERCLOS), and subjective sleepiness/fatigue measures (Stanford Sleepiness Scale, Samn-Perelli Fatigue Scale, and the Karolinska Sleepiness Scale, please refer to 2.1.3, 2.1.4).

SDLP is the standard deviation of the lateral position within a certain time interval, which is calculated with reference to the axis of the lane occupied by the participant’s vehicle. It is a measure of spatial/temporal variability of lane-keeping, and has been shown to be a valid tool to objectively assess driving fatigue (Åkerstedt et al., 2010, Vinckenbosch et al., 2020, Wang and Xu, 2016). As fatigue increases and alertness decreases, drivers tend to detect lane deviation more slowly and need larger steering wheel movements to correct their trajectory, which results in wider oscillation with respect to the lane axis (Thiffault & Bergeron, 2003). Therefore, higher SDLP values correspond to a higher level of driving fatigue.

PERCLOS (PERcent of eye CLOsure) is defined as the percentage of time of eyelid closure above a fixed threshold, within a certain time interval. It reflects slow eyelid closures rather than blinks, and is widely used as a drowsiness metric and to monitor alertness (Mallis and Dinges, 2004, Sahayadhas et al., 2012, Wierwille and Ellsworth, 1994). In line with most of the literature (McDonald et al., 2018, Van Loon et al., 2015), in this study a conservative 80% PERCLOS threshold was utilized, and calculated as percentage of the time spent with more than 80% of the pupil covered for each time interval, with reference to the detected maximum opening of each participant. Higher values of PERCLOS correspond to a higher level of driving fatigue.

For the present study, both SDLP and PERCLOS were aggregated within 5-minute intervals. In other words, the 50-minute experiment was discretized into ten 5-minute bins, and SDLP and PERCLOS computed within each bin.

### 2.3 Statistical analyses

The statistical analyses were conducted with R (version 4.0.4, R Core Team (2021), 2021).

Separate analyses were performed on the experimental group for each of the five dependent variables considered (Section 2.2): SDLP, PERCLOS, SSS, SPF, and KSS.

For the scalar dependent variables (SDLP and PERCLOS) linear mixed-effect models (LMMs) were used, considering as fixed factors Trial (2 levels, “pre-DST”, “post-DST”), and Time (10 levels, corresponding to the 10 5-minute bins in which the experiment was discretized), and as a random factor the ID of each participant. The factor Time was considered a marker of the passive TR component of driving fatigue (which relates to the monotonous driving task) and the factor Trial of the SR component (which relates to DST).

Successively, several sleep covariates (and their interaction with the factor Trial) were considered. The multivariate models were obtained with a backward elimination approach, progressively excluding nonsignificant covariates.

As for the historical cohort, the dependent variable SDLP was analysed with a similar approach, considering as fixed factors Trial (2 levels, “Trial 1”, “Trial 2”), and Time (10 levels, as for the experimental group), and as a random factor the ID of each participant. In this case, the factor Trial was considered as a marker of learning effect/familiarization with the driving scenario.

LMM was chosen due to its suitability to repeated-measures designs and its ability to handle missing data (e.g., some 5-minute PERCLOS values were discarded due to temporary eye tracker malfunction, please refer to Section 3.2). As for statistical inference, degrees of freedom for the reported *F*-statistics were computed by the Satterthwaite approximation, which does not assume equal variances (Kuznetsova et al., 2017, Satterthwaite, 1946). We refrained from performing statistical inference directly on LMM parameter estimates, as such an approach is considered less efficient in controlling for Type I errors, and interpretation becomes challenging when dealing with factors with several levels and interactions (Singmann & Kellen, 2019). Therefore, for brevity, parameter estimates are not reported.

To estimate LMMs, the package “lme4” (Bates, Mächler, Bolker, & Walker, 2015) was used, with the packages “lmerTest” (Kuznetsova et al., 2017) and “emmeans” (Lenth, Singmann, Love, Buerkner, & Herve, 2020) being adopted for *post hoc* analyses on the models.

Prior to model fitting, outliers were identified and investigated. Identification was carried out by using the boxplot method (Tukey, 1977), with separate boxplots built considering the different levels of the two main factors (Trial and Time). Outliers were then manually checked to verify whether they were the product of instrument malfunction or aberrant driving behaviour and removed if that was the case.

The assumption of normality of LMM residuals was checked using QQplot diagnostic. Despite LMMs being considered robust to violations of distributional assumptions (Schielzeth et al., 2020), for additional robustness we opted for applying a Box-Cox transformation of the dependent variable prior to model fitting when the assumption was not met (Box & Cox, 1964). For ease of interpretation, in Section 3 all reported marginal means and marginal trends were back-transformed; thus, the marginal trends illustrated in Fig. 4 and Fig. 6 are not linear.

For ordinal dependent variables (SSS, SPF, KSS) cumulative link mixed-effect models (CLMMs) were used (package “ordinal”) (Christensen, 2018), with a structure similar to the previously described LMMs, i.e., considering as fixed factors Trial and Time, and as a random factor the participant ID. Regarding the factor Time, this had 2 levels for the dependent variables SSS and SPF (“beginning”, “end”) and 5 levels for the variable KSS (each of the five self-evaluations performed every 9 min during the driving task).

The significance level was set at *α* = 0.05; *p*-values between 0.05 and 0.075 were reported as marginally significant.

## 3 Results

### 3.1 Driving-based measurement of fatigue

#### 3.1.1 Preliminary analyses

Before performing statistical analysis on SDLP values, data outliers were investigated. All outliers were then manually checked to ascertain that they were not caused by simulator malfunction or other technical problems. Since this was not the case, none of them was excluded from the analysis. Some of these outliers were caused by road departure events, defined as the vehicle exiting the carriageway with at least one wheel. Seven were recorded, 4 in the first trial, 3 in the second one; in each of these cases, drivers were able to quickly recover and return within the carriageway. We opted to keep the SDLP intervals containing these events, as they represented “extreme” situations of poor lateral control of the vehicle, and no abnormal driving behaviour (e.g., losing control or stopping the vehicle) was observed as a result. No collisions with other vehicles were recorded during the experiment.

An initial LMM was fitted considering the original values of SDLP. However, residual analysis showed a notable deviation from the normality assumption. For this reason, a Box-Cox transformation was performed (with an optimal lambda of −0.5), and the LMM was re-fitted on the transformed data. It should be noted that no changes in factor significance were observed when comparing the LMMs with transformed/untransformed data; for brevity and robustness, only the results of the LMMs estimated on transformed data will be presented.

#### 3.1.2 Main model

An LMM was fitted considering only the two main factors (Trial and Time) and the random factor (participant ID). A significant effect of Time was observed, *F*(9,323) = 13.6, *p* <.001; this is evidence of passive TR fatigue, as lateral control worsened over the course of the monotonous driving task.

**Fig. 2:**
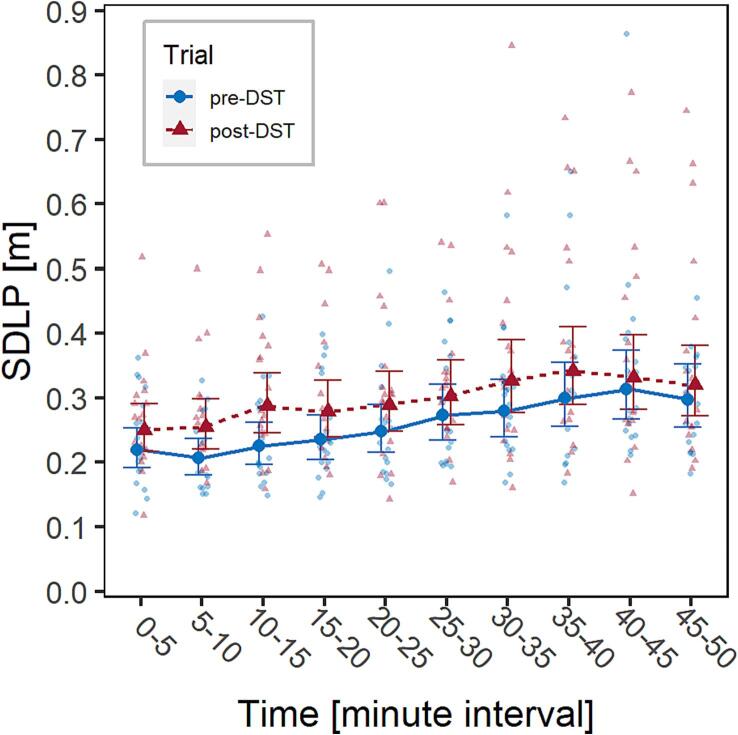
Visualization of the effect of the factors Trial (pre- and post-DST, marked in blue and red, respectively) and Time on SDLP in the experimental group. Solid dots represent marginal means, bars are 95% confidence intervals of marginal means, background dots are individual observations. (For interpretation of the references to colour in this figure legend, the reader is referred to the web version of this article.)

Interestingly, also the factor Trial had a significant effect on SDLP, *F*(1,323) = 43.5, *p* <.001, showing that driving performance worsened in the post-DST trial, in relation to SR fatigue (Fig. 2, Table S2 of Supplementary materials). The interaction between Trial and Time was not significant.

To investigate whether changes in SDLP between the two experimental trials might have been caused by an increased familiarity with the simulator and the experimental procedure, SDLP data from a control group (Section 2.1.1) were analysed.

The same procedure described in Section 3.1.1 was followed; no outliers were discarded and a Box-Cox transformation with lambda = 0.4 was performed.

**Fig. 3:**
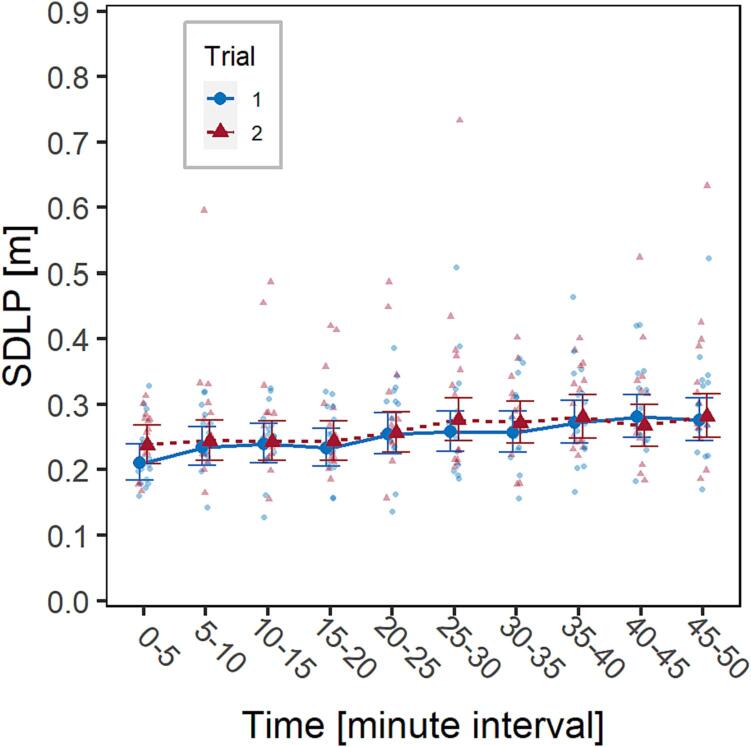
Visualization of the effect of the factors Trial (trial 1 and 2, marked in blue and red, respectively) and Time on SDLP in the control group. Solid dots represent marginal means, bars are 95% confidence intervals of marginal means, background dots are individual observations. (For interpretation of the references to colour in this figure legend, the reader is referred to the web version of this article.)

A significant effect of Time was observed, *F*(9,266) = 5.7, *p* <.001, with control group participants increasing their SDLP over the course of the driving task (passive TR fatigue), but without any effect of Trial, *F*(1,266) = 2.3, *p* = 0.13, nor of the interaction Trial*Time, *F*(9,266) = 0.5, *p* = 0.90 (Fig. 3, Table S3 of Supplementary materials). This implies that no familiarity effect on driving fatigue was observed in the control group, and that any significant difference in driving fatigue between the trials of the main experiment is reasonably to be imputed to the different experimental conditions, i.e., the Spring transition to DST, and therefore to SR driving fatigue.

#### 3.1.3 Multivariate model

Successively, several sleep-related covariates were considered for inclusion in the model. Descriptive statistics of such variables are reported in Table 1. The final model, which was obtained with a backward elimination procedure, contained the following covariates (and respective interaction with the factor Trial): Chronotype (nominal variable with 2 levels: morning and evening, from the Self-ME questionnaire), ESS (scalar, the ESS questionnaire score), habitual sleep duration during working days (SleepW, scalar, from the *µMCTQ* questionnaire), and sleep duration in the night before the trial (SleepNB, scalar, information collected before the start of each trial).

**Table 1:** Sleep characteristics of the experimental group participants. Sleep durations and midsleep are expressed in hh:mm format.

**Variable**	**Mean**	**SD**	**Min**	**Max**
ESS score	5.8	2.6	0	12
PSQI score	4.3	1.5	2	8
Sleep quality score	6.0	1.8	2	8
Habitual sleep duration (working days)	07:33	01:07	06:00	10:00
Habitual sleep duration (free days)	08:16	01:11	06:00	10:30
Midsleep (working days)	03:55	00:59	02:00	06:15
Midsleep (free days)	05:22	01:09	03:30	08:00
Social jet lag	01:27	00:59	−00:15	03:40
Sleep duration in the night before (pre-DST trial)	07:42	01:17	04:30	10:00
Sleep duration in the night before (post-DST trial)	07:48	01:23	04:30	09:30
Chronotype (morning/evening types)	9/9			

Other variables tested for inclusion but discarded from the final model were the PSQI questionnaire global score, STSQS questionnaire score, sleep duration during free days, midsleep during working days and free days, and social jetlag.

**Fig. 4:**
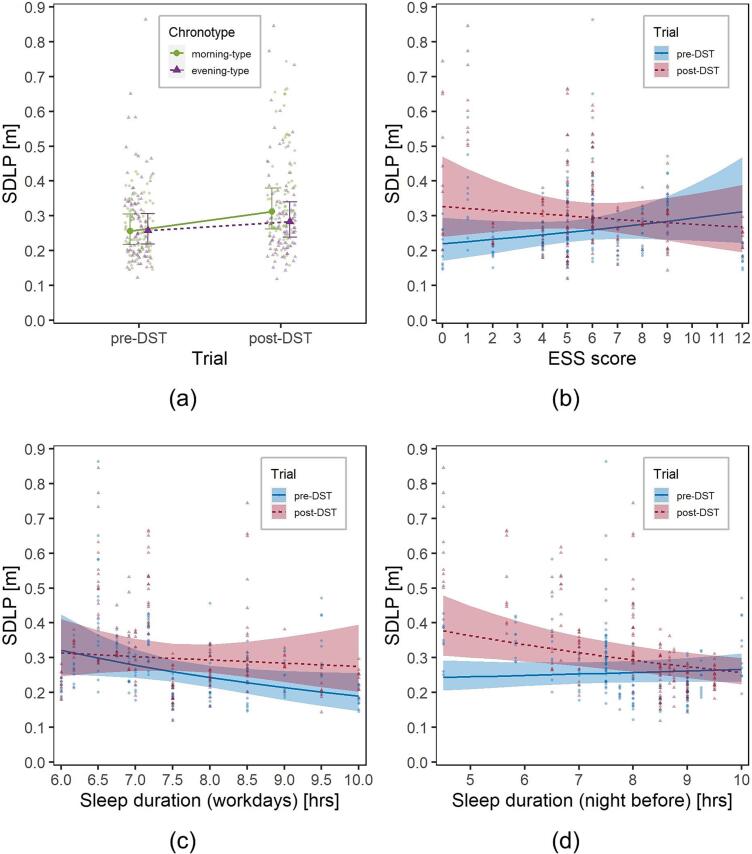
Visualization of the effects of the interactions on SDLP in the experimental group: (a) Trial (pre- and post-DST)*Chronotype (morning, in green, and evening, in purple) (b) Trial*ESS score; (c) Trial*SleepW; (d) Trial*SleepNB. Solid dots represent marginal means, lines are marginal trends; bars are 95% confidence intervals of marginal means; ribbons are 95% confidence intervals of marginal trends; background dots are individual observations. (For interpretation of the references to colour in this figure legend, the reader is referred to the web version of this article.)

The interaction between Trial and Chronotype had a significant effect on SDLP, *F*(1,317.08) = 6.1, *p* =.014 (Fig. 4a), with the DST affecting more severely morning-type participants.

Also the interactions of Trial with both ESS and SleepW were significant [*F*(1,320.90) = 29.7, *p* <.001; *F*(1,317.85) = 21.1, *p* <.001, respectively (Fig. 4b-c)], as participants with lower ESS values and higher habitual sleep duration tended to increase SDLP more.

On the other hand, those who slept less during the night before the trial were more severely affected by the switch to DST, as the interaction between Trial and SleepNB was significant, *F*(1,320.85) = 17.7, *p* <.001, Fig. 4d.

### 3.2 Physiological measurement of fatigue

To strengthen the findings of Section 3.1, the analyses were replicated considering a physiological approach for measuring driving fatigue: PERCLOS (please refer to Section 2.2).

The same procedure as in Section 3.1.1 was applied to detect outliers. In this case, the procedure was helpful to uncover some trials affected by technical malfunction of the eye tracker; as a result, data from two full and one partial driving simulation runs were excluded from the analysis. As the assumption of normality of the residuals was not met, a Box-Cox transformation was applied (with an optimal lambda of 0.2); again, no difference in statistical significance was observed between LMMs fitted with transformed and untransformed data, and the *p*-values reported in this section refer to the LMMs fitted on transformed data.

**Fig. 5:**
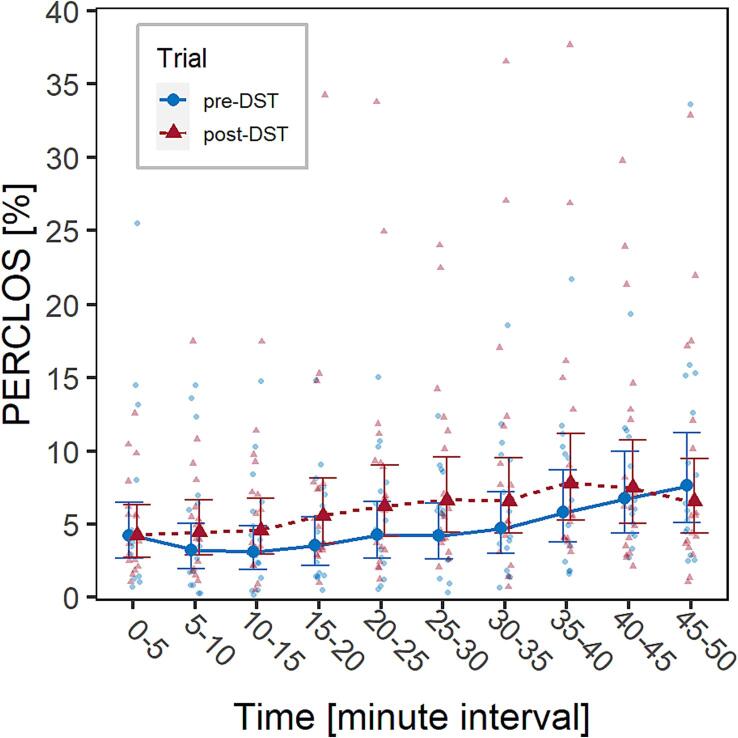
Visualization of the effect of the factors Trial (pre- and post-DST, marked in blue and red, respectively) and Time on PERCLOS in the experimental group. Solid dots represent marginal means, bars are 95% confidence intervals of marginal means, background dots are individual observations. (For interpretation of the references to colour in this figure legend, the reader is referred to the web version of this article.)

First, an LMM considering only the two main factors, Trial and Time, and their interaction was fitted. Time had a significant effect on PERCLOS, *F*(9,293.15) = 4.4, *p* <.001, the values of which tended to increase as the driving task progressed (Fig. 5, Table S4 of Supplementary materials). Trial was also significant, *F*(1,298.70) = 10.7, *p* <.001, with PERCLOS - and thus SR driving fatigue - being greater in the trial carried out after the switch to DST, consistent with what was observed for SDLP. The interaction between Trial and Time was not significant.

**Fig. 6:**
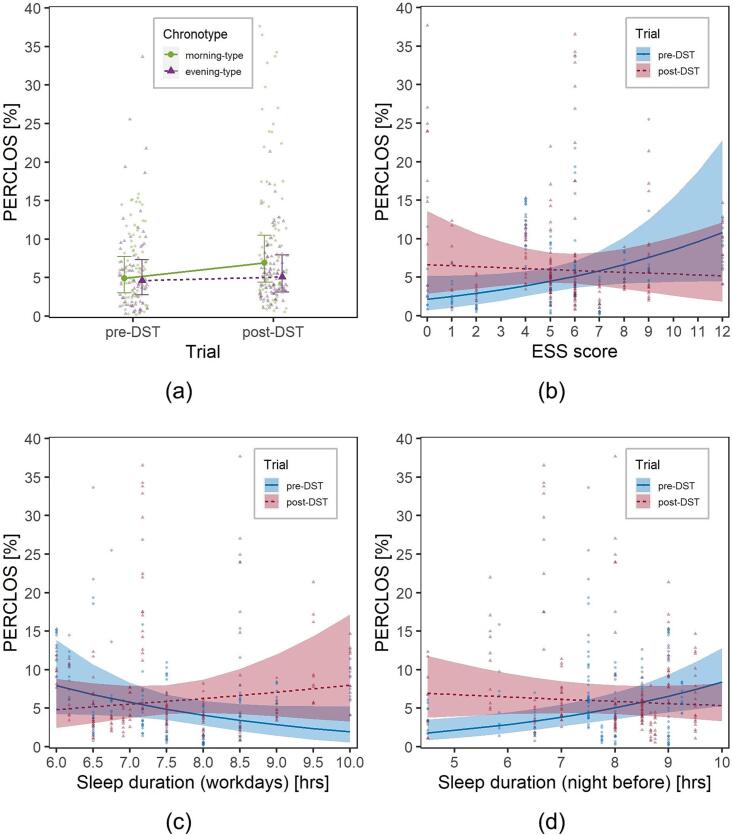
Visualization of the effects of the interactions on PERCLOS in the experimental group: (a) Trial (pre- and post-DST)*Chronotype (morning, in green, and evening, in purple) (b) Trial*ESS score; (c) Trial*SleepW; (d) Trial*SleepNB. Solid dots represent marginal means, lines are marginal trends; bars are 95% confidence intervals of marginal means; ribbons are 95% confidence intervals of marginal trends; background dots are individual observations. (For interpretation of the references to colour in this figure legend, the reader is referred to the web version of this article.)

Another LMM was fitted considering the same covariates and structure previously used for the analysis of SDLP (Section 3.1.3). Interaction between Trial and Chronotype was marginally significant, *F*(1,293.22) = 3.3, *p* =.072, while the interactions between Trial and ESS, SleepW and SleepNB were all significant [*F*(1,298.05) = 31.7, *p* <.001, *F*(1,291.41) = 36.4, *p* <.001, *F*(1,293.79) = 24.0, *p* <.001, respectively]. Fig. 6 can be used for the interpretation of these interactions, which are all consistent with what was observed in Section 3.1. In particular, drivers most affected by DST (i.e., with higher PERCLOS values) were characterized by morning chronotype, lower values of ESS, longer habitual sleep duration and shorter sleep duration in the night before the trial.

### 3.3 Subjective measurements of fatigue

To investigate whether there was correspondence between the observed objective measurements of driving fatigue and participants’ perception, several subjective measures of fatigue were recorded and analysed.

SSS and SPF self-reported questionnaires were administered before and after the driving task in both trials; the former evaluates sleepiness, the latter fatigue (please refer to 2.1.3, 2.1.4). The effects of Trial and Time (2 levels, “beginning” and “end” of the driving task) on the SSS and SPF scores were evaluated by fitting CLMMs.

**Fig. 7:**
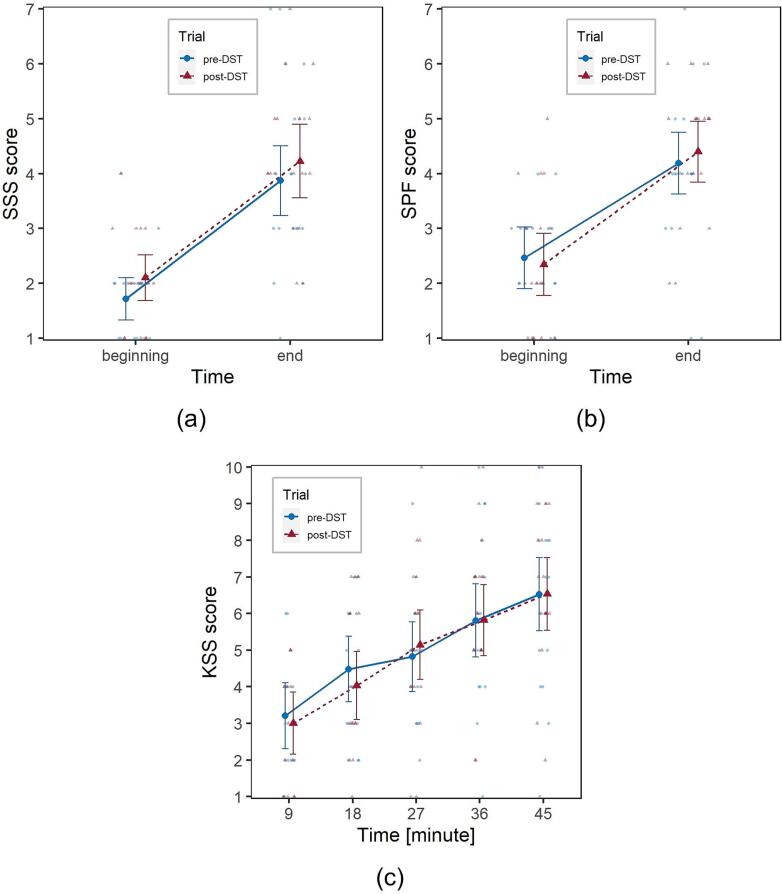
Visualization of the effect of the factors Trial (pre- and post-DST, marked in blue and red, respectively) and Time on: (a) SSS score; (b) SPF score; (c) KSS score. Solid dots represent marginal means, bars are 95% confidence intervals of marginal means, background dots are individual observations. (For interpretation of the references to colour in this figure legend, the reader is referred to the web version of this article.)

For both measures, Time had a significant effect, (χ^2^_1_ = 52.3, *p* <.001 on SSS; χ^2^_1_ = 34.7, *p* <.001 on SPF), meaning that participants perceived an increase in sleepiness and fatigue after the completion of the driving task. By contrast, Trial did not show any significant effect (χ^2^_1_ = 3.0, *p* =.084 on SSS; χ^2^_1_ = 0.03, *p* = 0.872 on SPF). The interaction Trial*Time was also not significant (Fig. 7a-b).

To track perceived fatigue during the task, participants had to verbally report a modified KSS score (range 1 to 10) five times (i.e., once every 9 min, please refer to Section 2.1.3). Another CLMM was estimated in order to assess the effect of Trial and Time (5 levels) on this score. Again, Time had a significant effect, χ^2^_4_ = 90.0, *p* <.001, participants progressively perceiving a decrease in their alertness throughout the driving task. However, consistent with what was observed for SSS and SPF, also in this case Trial and the interaction Trial*Time showed no significant effect, meaning that the significant increase in driving fatigue after DST (3.1, 3.2) was not perceived (Fig. 7c).

## 4 Discussion

### 4.1 Effect of DST transition on objective measurements of fatigue

The results of the present study document a significant effect of the DST transition on objective measurements of driving fatigue, with consistency between the two indicators analysed, i.e. SDLP and PERCLOS.

Participants tended to increase their values of SDLP and PERCLOS throughout the driving task both in the pre-DST and the post-DST tasks, as evidenced by a significant effect of the factor Time. This is an expected result, which had been observed also in the historical cohort, and indicates the presence of passive TR fatigue caused by driving in a monotonous highway environment, which was purposely built to induce mental underload.

The experimental group participants, returning for a second trial after the transition to DST, showed significantly higher values of SDLP and PERCLOS from the beginning and then throughout the driving task (factor Trial), indicating a higher level of fatigue, which can be reasonably attributed to DST itself. This interpretation is supported by the comparison with the historical cohort, in which no significant effect of the factor Trial was documented, thus excluding the presence of a learning effect/familiarization or other confounders related to the same task being repeated twice over a one-week period. Based on the sleep characteristics presented in Table 1, which indicates a consistent sleep duration among participants in the night prior to each trial, it is hypothesized that the observed effect on SR fatigue is likely due to DST-related circadian desynchrony and sleep quality, rather than a (potentially DST-unrelated) reduction in sleep duration.

In absolute terms, the marginal mean of SDLP was 0.256 m in the pre-DST trial and 0.296 m in the post-DST. This 4 cm increase is relevant also under a physical point of view, since, for reference, it is higher than the SDLP increase reported after the administration of 0.5 g/L alcohol (2.6 cm, van der Sluiszen et al., 2016, Vinckenbosch et al., 2020), and comparable to that reported after the administration of 10 mg of the sleep-inducing medication zolpidem (3.8 cm, Vinckenbosch et al., 2020). The marginal mean of PERCLOS increased from 4.59% of trial 1 to 5.90% of trial 2, which is comparable to the 1.66% increase observed in drivers performing a similar driving task after 24 h of total sleep deprivation (Jackson et al., 2016).

Of note, the increases observed in our experiment were higher than the 2.5 cm SDLP increase and the 0.43% PERCLOS increase observed in a previous experiment (same driving simulator and scenario) in partially sleep-deprived (5 h of sleep) participants (Cellini et al., 2023).

No interaction between the factors Time and Trial was observed. Participants indeed showed a higher fatigue since the early phases of the driving task and maintained it throughout. This is an interesting aspect with relevant safety implications, because it implies no relationship between passive TR fatigue and DST-induced SR fatigue. In other words, the negative effects on fatigue were evident since the very beginning of the trial, indicating that this is an issue that does not necessarily emerge only in mental underload situations. This is also consistent with the observations made in a previous DST study from our group, in which we observed a deterioration of driving performance in terms of reaction times, rule violations and lateral control over a relative short (less than 15 min) driving task post-DST (Orsini et al., 2022).

### 4.2 The modulating effect of drivers’ sleep characteristics

The results of the multivariate models suggest that the effect of the DST transition on SDLP and PERCLOS were modulated by the drivers’ sleep-wake characteristics.

Firstly, DST affected more severely morning-type participants. This is consistent with the experimental design expectations, since the driving sessions took place during the afternoon.

In addition, participants with lower ESS scores and longer habitual sleep duration increased their SDLP and PERCLOS more. This suggests that participants somewhat used to sleep deprivation and drowsiness were able to compensate for SR driving fatigue more efficiently than those with better ESS scores and longer habitual sleep duration.

Finally, people who slept less in the night prior to the post-DST session had significantly higher driving fatigue issues, further highlighting the relationship between DST and SR fatigue.

These results shed additional light on DST-related effects on driving fatigue: the most affected were morning-type participants (most likely in relation to the fact that trials were carried out in the afternoon), those who slept less during the night before the test and those who were not used to drowsiness and sleep deprivation. Besides being novel, the presented results also provide a flavour of the complexity of the effects of DST on daytime and specific task performance.

### 4.3 Effect of DST transition on subjective fatigue

It is interesting to compare the results of the objective measurements of fatigue discussed in Section 4.1 with those of subjectively perceived fatigue, with a view to understand whether drivers are aware of their increase in DST-related fatigue. The three measures analysed in Section 3.3, SSS, SPF and KSS, provide similar results and can be interpreted and discussed together.

The effect of the factor Time was significant, meaning that participants were able to perceive their increase in sleepiness and fatigue over the driving task, which is consistent with the objective fatigue measurements.

Interestingly though, no effect of the factor Trial was observed, which suggests that participants were unable to perceive the objective increase in their SR driving fatigue in the trial performed after the switch to DST. This may depend on either the size of the effect or on some degree of diminished insight of the participants into their actual performance due to circadian desynchrony and/or sleep deprivation (Martínez-Pérez, Palmero, Campoy, & Fuentes, 2020).

It is our impression that these results have relevant practical implications, especially as the observed objective increase in fatigue was not only meaningful from a statistical point of view, but also in practical terms (e.g., an increase of 4 cm in SDLP is comparable to the effect of hypnotics, according to the findings of Vinckenbosch et al., 2020), leading to relevant safety issues. The inability of participants to perceive this implies that they could not implement any compensatory or fatigue-coping strategy, and may be related to the observed increase in road crashes in the week following the switch to DST (Fritz et al., 2020).

## 5 Conclusion

This study investigated the effect of the Spring transition to DST on driving fatigue, by means of a driving simulator experiment. Fatigue was measured considering a wide range of both objective and subjective indices/metrics.

1. Participants exhibited an increase in SR driving fatigue during the post-DST trial, when considering both objective measurements, i.e., SDLP and PERCLOS. In absolute terms, the increase in fatigue as assessed by SDLP was greater than that previously documented for alcohol and comparable to that previously documented for a hypnotic (van der Sluiszen et al., 2016, Vinckenbosch et al., 2020); the increase in PERCLOS values was comparable to that of sleep-deprived drivers (Jackson et al., 2016). Both were higher than the increases observed in partially sleep-deprived drivers in the same simulator scenario (Cellini et al., 2023).
2. The effect of DST transition was more prominent in morning-type participants, in those who normally do not suffer from daytime sleepiness, who are used to longer habitual sleep duration and who slept less during the night prior to the trial. In brief, those affected were people less used to dealing with sleepiness while driving, and thus less experienced in applying fatigue-coping strategies. These drivers may represent a significant proportion of the general population, further supporting the hypothesis that the switch to DST can produce serious road safety issues.
3. Participants seemed substantially unaware of the worsening of their driving performance after DST, which is critical from a road safety perspective, as drivers are not enticed to implement compensatory fatigue-coping strategies. These findings seem worthy of further study, may contribute to the ongoing debate on the appropriateness of abolishing DST and may even become the subject of sensitising campaigns dedicated to informing the general population on these DST effects and how to prevent them.

The main limitation of this study is that, due to the strict time constraints of the experimental procedure, only young male Italian drivers were included, preventing investigation of other potentially relevant factors such as age, gender, culture, and location. In this sense, it can be considered as a pilot study. In addition, future research will be needed to evaluate the long-term effects of the Spring transition to DST, as here we focused exclusively on its short-term effects.

## Funding

This study was supported by a University of Padova STARS@UNIPD 2019 Consolidator Grant to SM, which also part-funded GG and LZ, and by the CINCHRON, EU Horizon 2020, Marie Sklodowska-Curie Initial Training Network grant (agreement N° 765937) to RC.

## CRediT authorship contribution statement

**Federico Orsini:** Conceptualization, Methodology, Software, Formal analysis, Investigation, Data curation, Writing – original draft, Writing – review & editing, Visualization. **Gianluca Giusti:** Methodology, Investigation, Data curation, Writing – review & editing. **Lisa Zarantonello:** Methodology, Data curation, Writing – review & editing. **Rodolfo Costa:** Conceptualization, Methodology, Supervision, Funding acquisition, Writing - original draft, Writing - review & editing. **Sara Montagnese:** Conceptualization, Methodology, Resources, Writing – original draft, Writing – review & editing, Supervision, Funding acquisition. **Riccardo Rossi:** Conceptualization, Methodology, Validation, Resources, Writing – review & editing, Supervision, Funding acquisition.

## Declaration of Competing Interest

The authors declare that they have no known competing financial interests or personal relationships that could have appeared to influence the work reported in this paper.

## Data Availability

Data will be made available on request.

## References

[b0005] Åkerstedt T., Ingre M., Kecklund G., Anund A., Sandberg D., Wahde M., Kronberg P. (2010). Reaction of sleepiness indicators to partial sleep deprivation, time of day and time on task in a driving simulator - The DROWSI project. Journal of Sleep Research.

[b0010] Åkerstedt T., Peters B., Anund A., Kecklund G. (2005). Impaired alertness and performance driving home from the night shift: A driving simulator study. Journal of Sleep Research.

[b0015] Alhola P., Polo-Kantola P. (2007). Sleep deprivation: Impact on cognitive performance. Neuropsychiatric Disease and Treatment.

[b0020] Alvaro P.K., Burnett N.M., Kennedy G.A., Min W.Y.X., McMahon M., Barnes M., Howard M.E. (2018). Driver education: Enhancing knowledge of sleep, fatigue and risky behaviour to improve decision making in young drivers. Accident; Analysis and Prevention.

[b0025] Bates D., Mächler M., Bolker B.M., Walker S.C. (2015). Fitting linear mixed-effects models using lme4. Journal of Statistical Software.

[b0030] Bayne A., Trivedi N., Liotta M., Siegfried A., Gaspar J., Carney C. (2022).

[b0035] Berry, R. B., & Wagner, M. H. (2014). Sleep Medicine Pearls, Sleep Medicine Pearls. Elsevier Inc. doi:10.1016/C2012-0-03542-9.

[b0040] Bioulac S., Micoulaud Franchi J.A., Arnaud M., Sagaspe P., Moore N., Salvo F., Philip P. (2017). Risk of motor vehicle accidents related to sleepiness at the wheel: A systematic review and meta-analysis. Sleep.

[b0045] Box G.E.P., Cox D.R. (1964). An Analysis of Transformations. Journal of the Royal Statistical Society, Series B.

[b0050] Boyle L.N., Tippin J., Paul A., Rizzo M. (2008). Driver performance in the moments surrounding a microsleep. Transportation Research Part F: Traffic Psychology and Behaviour.

[b0055] Buysse D.J., Reynolds C.F., Monk T.H., Berman S.R., Kupfer D.J. (1989). The Pittsburgh sleep quality index: A new instrument for psychiatric practice and research. Psychiatry Research.

[b0060] CAA SRG (2005). Aircrew Fatigue: A Review of Research Undertaken on Behalf of the UK Civil Aviation Authority www.caa.co.uk Safety Regulation Group.

[b0065] Cai A.W.T., Manousakis J.E., Singh B., Kuo J., Jeppe K.J., Francis-Pester E., Anderson C. (2021). On-road driving impairment following sleep deprivation differs according to age. Scientific Reports.

[b0070] Carey R.N., Sarma K.M. (2017). Impact of daylight saving time on road traffic collision risk: A systematic review. BMJ Open.

[b0075] Cellini N., Bruno G., Orsini F., Vidotto G., Gastaldi M., Rossi R., Tagliabue M. (2023). The Effect of Partial Sleep Deprivation and Time-on-Task on Young Drivers’ Subjective and Objective Sleepiness. International Journal of Environmental Research and Public Health.

[b0080] Christensen, R. H. B. (2018). Cumulative link models for ordinal regression with the R Package ordinal.

[b0085] Coate D., Markowitz S. (2004). The effects of daylight and daylight saving time on US pedestrian fatalities and motor vehicle occupant fatalities. Accident; Analysis and Prevention.

[b0090] Curcio G., Tempesta D., Scarlata S., Marzano C., Moroni F., Rossini P.M., De Gennaro L. (2013). Validity of the Italian Version of the Pittsburgh Sleep Quality Index (PSQI). Neurological Sciences.

[b0095] Du H., Zhao X., Zhang X., Zhang Y., Rong J. (2015). Effects of Fatigue on Driving Performance Under Different Roadway Geometries: A Simulator Study. Traffic Injury Prevention.

[b0100] Ellis W.A., FitzGibbon S.I., Barth B.J., Niehaus A.C., David G.K., Taylor B.D., Wilson R.S. (2016). Daylight saving time can decrease the frequency of wildlife-vehicle collisions. Biology Letters.

[b0105] Observatory E.R.S. (2021). Road Safety Thematic Report-Fatigue. European Road Safety. Observatory.

[b0110] Ferrazzi E., Romualdi C., Ocello M., Frighetto G., Turco M., Vigolo S., Montagnese S. (2018). Changes in Accident & Emergency Visits and Return Visits in Relation to the Enforcement of Daylight Saving Time and Photoperiod. Journal of Biological Rhythms.

[b0115] Fritz J., VoPham T., Wright K.P., Vetter C. (2020). A Chronobiological Evaluation of the Acute Effects of Daylight Saving Time on Traffic Accident Risk. Current Biology.

[b0120] Gastaldi, M., Rossi, R., Hadas, Y., Fasan, D., Keren, N., & Mulatti, C. (2016). Caffeinated Chewing Gum as Countermeasure to Drivers’ Passive Task-Related Fatigue Caused by Monotonous Roadway: 2602, 26–34. doi: 10.3141/2602-04.

[b0125] Ghotbi N., Pilz L.K., Winnebeck E.C., Vetter C., Zerbini G., Lenssen D., Roenneberg T. (2020). The µMCTQ: An Ultra-Short Version of the Munich ChronoType Questionnaire. Journal of Biological Rhythms.

[b0130] Gimeno P.T., Cerezuela G.P., Montañés M.C. (2006). On the concept and measurement of driver drowsiness, fatigue and inattention: Implications for countermeasures. International Journal of Vehicle Design.

[b0135] Gonçalves M., Amici R., Lucas R., Åkerstedt T., Cirignotta F., Horne J., Grote L. (2015). Sleepiness at the wheel across Europe: A survey of 19 countries. Journal of Sleep Research.

[b0140] Hamid M., Samuel S., Borowsky A., Horrey W.J., Fisher D.L. (2016). Evaluation of Training Interventions to Mitigate Effects of Fatigue and Sleepiness on Driving Performance. Transportation Research Record.

[b0145] Hilditch C.J., Dorrian J., Centofanti S.A., Van Dongen H.P., Banks S. (2017). Sleep inertia associated with a 10-min nap before the commute home following a night shift: A laboratory simulation study. Accident; Analysis and Prevention.

[b0150] Hoddes E., Zarcone V., Smythe H., Phillips R., Dement W.C. (1973). Quantification of Sleepiness: A New Approach. Psychophysiology.

[b0155] Huang A., Levinson D. (2010). The effects of daylight saving time on vehicle crashes in Minnesota. Journal of Safety Research.

[b0160] Jackson M.L., Raj S., Croft R.J., Hayley A.C., Downey L.A., Kennedy G.A., Howard M.E. (2016). Slow eyelid closure as a measure of driver drowsiness and its relationship to performance. Traffic Injury Prevention.

[b0165] Johns M.W. (1991). A new method for measuring daytime sleepiness: The Epworth sleepiness scale. Sleep.

[b0170] Jongen S., Perrier J., Vuurman E.F., Ramaekers J.G., Vermeeren A. (2015). Sensitivity and validity of psychometric tests for assessing driving impairment: Effects of sleep deprivation. PLoS One1.

[b0175] Kuznetsova A., Brockhoff P.B., Christensen R.H.B. (2017). lmerTest Package: Tests in Linear Mixed Effects Models. Journal of Statistical Software.

[b0180] Lahti T., Nysten E., Haukka J., Sulander P., Partonen T. (2010). Daylight saving time transitions and road traffic accidents. Journal of Environmental and Public Health.

[b0185] Lenth, R., Singmann, H., Love, J., Buerkner, P., Herve, M., 2020. emmeans: estimated marginal means. R package version 1.4. 4. Am. Stat.

[b0190] Li R., Su W., Lu Z. (2017). Physiological signal analysis for fatigue level of experienced and inexperienced drivers. Traffic Injury Prevention.

[b0195] Lyznicki J.M., Doege T.C., Davis R.M., Williams M.A., Bresolin L.B. (1998). Sleepiness, driving, and motor vehicle crashes. Journal of the American Medical Association.

[b0200] Mallis M.M., Dinges D.F. (2004). Handbook of Human Factors and Ergonomics Methods.

[b0205] Martínez-Pérez V., Palmero L.B., Campoy G., Fuentes L.J. (2020). The role of chronotype in the interaction between the alerting and the executive control networks. Scientific Reports.

[b0210] Martiniuk A.L.C., Senserrick T., Lo S., Williamson A., Du W., Grunstein R.R., Ivers R.Q. (2013). Sleep-deprived young drivers and the risk for crash the drive prospective cohort study. JAMA Pediatrics.

[b0215] May J.F., Baldwin C.L. (2009). Driver fatigue: The importance of identifying causal factors of fatigue when considering detection and countermeasure technologies. Transportation Research Part F: Traffic Psychology and Behaviour.

[b0220] McDonald A.D., Lee J.D., Schwarz C., Brown T.L. (2018). A contextual and temporal algorithm for driver drowsiness detection. Accident; Analysis and Prevention.

[b0225] Montagnese S., Middleton B., Skene D.J., Morgan M.Y. (2009). Sleep-wake patterns in patients with cirrhosis: All you need to know on a single sheet. A simple sleep questionnaire for clinical use. Journal of Hepatology.

[b0230] Orsini F., Gecchele G., Gastaldi M., Rossi R. (2019). Collision prediction in roundabouts: A comparative study of extreme value theory approaches. Transp. A Transp. Sci..

[b0235] Orsini F., Zarantonello L., Costa R., Rossi R., Montagnese S. (2022). Driving simulator performance worsens after the Spring transition to Daylight Saving Time. iScience.

[b0240] Philip P., Sagaspe P., Moore N., Taillard J., Charles A., Guilleminault C., Bioulac B. (2005). Fatigue, sleep restriction and driving performance. Accident; Analysis and Prevention.

[b0245] Phillips, R. O. (2014). What is fatigue and how does it affect the safety performance of human transport operators? Institute of Transport Economics (TØI), Oslo.

[b0250] R Core Team (2021). R: A Language and Environment for Statistical Computing.

[b0255] Robb D., Barnes T. (2018). Accident rates and the impact of daylight saving time transitions. Accident; Analysis and Prevention.

[b0260] Roenneberg T., Winnebeck E.C., Klerman E.B. (2019). Daylight saving time and artificial time zones - A battle between biological and social times. Frontiers in Physiology.

[b0265] Roenneberg T., Wirz-Justice A., Merrow M. (2003). Life between clocks: Daily temporal patterns of human chronotypes. Journal of Biological Rhythms.

[b0270] Rossi R., Gastaldi M., Biondi F., Mulatti C. (2012). Evaluating the impact of processing spoken words on driving. Transportation Research Record.

[b0275] Rossi R., Gastaldi M., Gecchele G., Biondi F., Mulatti C. (2014). Traffic-calming measures affecting perceived speed in approaching bends: On-field validated virtual environment. Transportation Research Record.

[b0280] Rossi R., Meneguzzer C., Orsini F., Gastaldi M. (2020). Gap-acceptance behavior at roundabouts: Validation of a driving simulator environment using field observations. Transportation Research Procedia.

[b0285] Rossi R., Orsini F., Tagliabue M., Di Stasi L.L., De Cet G., Gastaldi M. (2021). Evaluating the impact of real-time coaching programs on drivers overtaking cyclists. Transportation Research Part F: Traffic Psychology and Behaviour.

[b0290] Rubaltelli E., Manicardi D., Orsini F., Mulatti C., Rossi R., Lotto L. (2021). How to nudge drivers to reduce speed: The case of the left-digit effect. Transportation Research Part F: Traffic Psychology and Behaviour.

[b0295] Sahayadhas A., Sundaraj K., Murugappan M. (2012). Detecting driver drowsiness based on sensors: A review. Sensors (Switzerland).

[b0300] Samn, S., Perelli, L. (1982). Estimating Aircrew Fatigue: A Technique with Application to Airlift Operations. USAF Sch. Med. 26.

[b0305] Sandberg D., Anund A., Fors C., Kecklund G., Karlsson J.G., Wahde M., Åkerstedt T. (2011). The characteristics of sleepiness during real driving at night - A study of driving performance, physiology and subjective experience. Sleep.

[b0310] Satterthwaite F.E. (1946). An Approximate Distribution of Estimates of Variance Components. Biometrics Bulletin.

[b0315] Schielzeth H., Dingemanse N.J., Nakagawa S., Westneat D.F., Allegue H., Teplitsky C., Araya-Ajoy Y.G. (2020). Robustness of linear mixed-effects models to violations of distributional assumptions. Methods in Ecology and Evolution.

[b0320] Singh R., Sood R., Graham D.J. (2022). Road traffic casualties in Great Britain at daylight savings time transitions: A causal regression discontinuity design analysis. BMJ Open.

[b0325] Singmann H., Kellen D. (2019). An introduction to mixed models for experimental psychology. New Methods in Cognitive Psychology.

[b0330] Sullivan J.M., Flannagan M.J. (2002). The role of ambient light level in fatal crashes: Inferences from daylight saving time transitions. Accident; Analysis and Prevention.

[b0335] Thiffault P., Bergeron J. (2003). Monotony of road environment and driver fatigue: A simulator study. Accident; Analysis and Prevention.

[b0340] Tukey J. (1977).

[b0345] Turco M., Corrias M., Chiaromanni F., Bano M., Salamanca M., Caccin L., Montagnese S. (2015). The self-morningness/eveningness (Self-ME): An extremely concise and totally subjective assessment of diurnal preference. Chronobiology International.

[b0350] van der Sluiszen N.N.J.J.M., Vermeeren A., Jongen S., Theunissen E.L., van Oers A.C.M., Van Leeuwen C.J., Ramaekers J.G. (2016). On-the-road driving performance after use of the antihistamines mequitazine and l-mequitazine, alone and with alcohol. Psychopharmacology.

[b0355] Van Loon R.J., Brouwer R.F.T., Martens M.H. (2015). Drowsy drivers’ under-performance in lateral control: How much is too much? Using an integrated measure of lateral control to quantify safe lateral driving. Accident; Analysis and Prevention.

[b0360] Vignatelli L., Plazzi G., Barbato A., Ferini-Strambi L., Manni R., Pompei F., Gigli G.L. (2003). Italian version of the Epworth sleepiness scale: External validity. Neurological Sciences.

[b0365] Vinckenbosch F.R.J., Vermeeren A., Verster J.C., Ramaekers J.G., Vuurman E.F. (2020). Validating lane drifts as a predictive measure of drug or sleepiness induced driving impairment. Psychopharmacology.

[b0370] Wang X., Xu C. (2016). Driver drowsiness detection based on non-intrusive metrics considering individual specifics. Accident; Analysis and Prevention.

[b0375] Wierwille W.W., Ellsworth L.A. (1994). Evaluation of driver drowsiness by trained raters. Accident; Analysis and Prevention.

[b0380] World Medical Association, 2013. World Medical Association declaration of Helsinki: Ethical principles for medical research involving human subjects. JAMA - J. Am. Med. Assoc. doi: 10.1001/jama.2013.281053. 19886379

[b0385] Wörle J., Metz B., Steinborn M.B., Huestegge L., Baumann M. (2021). Differential effects of driver sleepiness and sleep inertia on driving behavior. Transportation Research Part F: Traffic Psychology and Behaviour.

[b0390] Zhang G., Yau K.K.W., Zhang X., Li Y. (2016). Traffic accidents involving fatigue driving and their extent of casualties. Accident; Analysis and Prevention.

[b0395] Zhang H., Yan X., Wu C., Qiu T. (2014). Effect of circadian rhythms and driving duration on fatigue level and driving performance of professional drivers. Transportation Research Record.

[b0400] Zhou R., Li Y. (2022). Traffic crash changes following transitions between daylight saving time and standard time in the United States: New evidence for public policy making. Journal of Safety Research.

