# Corrigendum to “Driving fatigue increases after the spring transition to daylight saving time in young male drivers: A pilot study” [Transport. Res. Part F: Traffic Psychol. Behav. 99 (2023) 83–97]

**DOI:** 10.1016/j.trf.2024.03.016

**Published:** 2024-10

**Authors:** Federico Orsini, Gianluca Giusti, Lisa Zarantonello, Rodolfo Costa, Sara Montagnese, Riccardo Rossi

**Affiliations:** a Department of Civil, Environmental and Architectural Engineering, University of Padua, Padua, Italy; b Mobility and Behavior Research Center – MoBe, University of Padua, Padua, Italy; c Department of General Psychology, University of Padua, Padua, Italy; d Department of Medicine, University of Padua, Padua, Italy; e Chronobiology Section, Faculty of Health and Medical Sciences, University of Surrey, Guildford, United Kingdom; f Institute of Neuroscience, National Research Council (CNR), Padua, Italy; g Department of Biology, University of Padua, Padua, Italy

The authors regret that a founder was missing in the “Funding” Section. The “Funding” Section should contain the following text:

***Funding***

*This study was supported by a University of Padova STARS@UNIPD 2019 Consolidator Grant to SM, which also part-funded GG and LZ, by the CINCHRON, EU Horizon 2020, Marie Sklodowska-Curie Initial Training Network grant (agreement N° 765937) to RC, and by the Telethon Foundation (grant GGP15041) to RC.*

The authors would like to apologise for any inconvenience caused.

