# Supplementary material for: Driving fatigue increases after the Spring transition to Daylight Saving Time in young male drivers: A pilot study
Source: Transp Res Part F Traffic Psychol Behav. 2023 Nov;99:83–97. doi: 10.1016/j.trf.2023.10.014 (PMC10988525; doi:10.1016/j.trf.2023.10.014)
Supplement: Supplementary data 1 [file mmc1.docx]

# Supplementary material

Table S1. Sociodemographic, driving and sleep characteristics of the Control and Experimental groups (standard deviation in round brackets)

| **Variable** | **Control group** | **Experimental group** |
| --- | --- | --- |
| N | 15 | 18 |
| Age [years] | 24.2 (3.4) | 24.2 (2.9) |
| Gender | 100% males | 100% males |
| Profession | 87% students, 13% workers | 89% students, 11% workers |
| Driving experience [years] | 5.8 (2.4) | 5.8 (3.0) |
| Annual mileage [km] | 11,386 (8,040) | 10,468 (12,690) |
| Epworth Sleepiness Scale | 6.4 (3.3) | 5.8 (2.6) |

Table S2. Marginal means, standard error and 95% confidence interval of SDLP, expressed in meters, for the experimental group

| **Time** | **Trial** | **Mean** | **SE** | **95% CI** |
| --- | --- | --- | --- | --- |
| 0-5 | pre-DST | 0.219 | 0.0152 | [0.192;0.254] |
| 5-10 | pre-DST | 0.206 | 0.0139 | [0.181;0.237] |
| 10-15 | pre-DST | 0.226 | 0.0159 | [0.197;0.262] |
| 15-20 | pre-DST | 0.236 | 0.0169 | [0.205;0.274] |
| 20-25 | pre-DST | 0.249 | 0.0183 | [0.215;0.290] |
| 25-30 | pre-DST | 0.273 | 0.0211 | [0.235;0.321] |
| 30-35 | pre-DST | 0.280 | 0.0219 | [0.240;0.329] |
| 35-40 | pre-DST | 0.300 | 0.0243 | [0.256;0.355] |
| 40-45 | pre-DST | 0.314 | 0.0261 | [0.268;0.374] |
| 45-50 | pre-DST | 0.298 | 0.024 | [0.255;0.353] |
| 0-5 | post-DST | 0.250 | 0.0185 | [0.216;0.292] |
| 5-10 | post-DST | 0.255 | 0.0191 | [0.221;0.298] |
| 10-15 | post-DST | 0.287 | 0.0228 | [0.246;0.340] |
| 15-20 | post-DST | 0.279 | 0.0217 | [0.239;0.328] |
| 20-25 | post-DST | 0.289 | 0.023 | [0.248;0.342] |
| 25-30 | post-DST | 0.303 | 0.0247 | [0.259;0.360] |
| 30-35 | post-DST | 0.327 | 0.0276 | [0.277;0.390] |
| 35-40 | post-DST | 0.342 | 0.0296 | [0.289;0.411] |
| 40-45 | post-DST | 0.332 | 0.0284 | [0.282;0.398] |
| 45-50 | post-DST | 0.320 | 0.0268 | [0.272;0.382] |

Table S3. Marginal means, standard error and 95% confidence interval of SDLP, expressed in meters, for the control group

| **Time** | **Trial** | **Mean** | **SE** | **95% CI** |
| --- | --- | --- | --- | --- |
| 0-5 | 1 | 0.210 | 0.0138 | [0.184;0.239] |
| 5-10 | 1 | 0.235 | 0.0147 | [0.206;0.266] |
| 10-15 | 1 | 0.240 | 0.0149 | [0.211;0.271] |
| 15-20 | 1 | 0.233 | 0.0147 | [0.205;0.264] |
| 20-25 | 1 | 0.255 | 0.0155 | [0.225;0.287] |
| 25-30 | 1 | 0.258 | 0.0156 | [0.228;0.290] |
| 30-35 | 1 | 0.258 | 0.0156 | [0.228;0.290] |
| 35-40 | 1 | 0.272 | 0.0161 | [0.241;0.306] |
| 40-45 | 1 | 0.281 | 0.0164 | [0.250;0.316] |
| 45-50 | 1 | 0.276 | 0.0162 | [0.245;0.310] |
| 0-5 | 2 | 0.238 | 0.0148 | [0.209;0.269] |
| 5-10 | 2 | 0.244 | 0.0151 | [0.215;0.276] |
| 10-15 | 2 | 0.243 | 0.015 | [0.214;0.274] |
| 15-20 | 2 | 0.243 | 0.015 | [0.214;0.275] |
| 20-25 | 2 | 0.257 | 0.0155 | [0.227;0.289] |
| 25-30 | 2 | 0.276 | 0.0162 | [0.244;0.310] |
| 30-35 | 2 | 0.272 | 0.0161 | [0.241;0.305] |
| 35-40 | 2 | 0.281 | 0.0164 | [0.249;0.315] |
| 40-45 | 2 | 0.267 | 0.0159 | [0.236;0.300] |
| 45-50 | 2 | 0.282 | 0.0164 | [0.250;0.316] |

Table S4. Marginal means, standard error and 95% confidence interval of PERCLOS, for the experimental group

| **Time** | **Trial** | **Mean** | **SE** | **95% CI** |
| --- | --- | --- | --- | --- |
| 0-5 | pre-DST | 4.25 | 0.954 | [2.66;6.51] |
| 5-10 | pre-DST | 3.20 | 0.762 | [1.95;5.03] |
| 10-15 | pre-DST | 3.11 | 0.743 | [1.89;4.89] |
| 15-20 | pre-DST | 3.53 | 0.824 | [2.17;5.50] |
| 20-25 | pre-DST | 4.29 | 0.962 | [2.69;6.57] |
| 25-30 | pre-DST | 4.21 | 0.948 | [2.64;6.46] |
| 30-35 | pre-DST | 4.73 | 1.040 | [3.00;7.18] |
| 35-40 | pre-DST | 5.82 | 1.228 | [3.76;8.70] |
| 40-45 | pre-DST | 6.75 | 1.382 | [4.42;9.97] |
| 45-50 | pre-DST | 7.68 | 1.533 | [5.08;11.24] |
| 0-5 | post-DST | 4.22 | 0.892 | [2.72;6.32] |
| 5-10 | post-DST | 4.46 | 0.933 | [2.89;6.65] |
| 10-15 | post-DST | 4.56 | 0.950 | [2.96;6.79] |
| 15-20 | post-DST | 5.54 | 1.110 | [3.66;8.13] |
| 20-25 | post-DST | 6.22 | 1.218 | [4.15;9.05] |
| 25-30 | post-DST | 6.63 | 1.282 | [4.45;9.61] |
| 30-35 | post-DST | 6.58 | 1.274 | [4.41;9.54] |
| 35-40 | post-DST | 7.80 | 1.460 | [5.30;11.17] |
| 40-45 | post-DST | 7.48 | 1.411 | [5.06;10.74] |
| 45-50 | post-DST | 6.55 | 1.269 | [4.38;9.49] |
